# Gender Differences and the Influence of Body Composition on Land and Pool-Based Assessments of Anaerobic Power and Capacity

**DOI:** 10.3390/ijerph19137902

**Published:** 2022-06-28

**Authors:** Jacquelyn N. Zera, Elizabeth F. Nagle, Emma Connell, Erin Curtin, Wilmina Marget, Anna P. Simonson, Takashi Nagai, John Abt, Scott Lephart

**Affiliations:** 1 Department of Exercise Science and Sport Studies, John Carroll University, University Heights, OH 44118, USA; asimonson@jcu.edu; 2 Department of Health and Physical Activity, University of Pittsburgh, Pittsburgh, PA 15260, USA; nagle@pitt.edu; 3 Veteran Affairs Puget Sound Medical Center, Seattle, WA 98108, USA; connell.emma.t@gmail.com; 4 Department of Mathematics and Computer Science, John Carroll University, University Heights, OH 44118, USA; ecurtin18@jcu.edu (E.C.); billiemarget@gmail.com (W.M.); 5 Sports Medicine Center, Mayo Clinic, Rochester, MN 55902, USA; takashi.nagai.civ@mail.mil; 6 Children’s Health Andrews Institute for Orthopaedics and Sports Medicine, Plano, TX 75024, USA; john.abt@childrens.com; 7 College of Health Sciences, University of Kentucky, Lexington, KY 40506, USA; scott.lephart@uky.edu

**Keywords:** swimming, gender, force production

## Abstract

Consistent differences between males and females have been shown in land-based measurements of anaerobic power and capacity. However, these differences have not been investigated for a tethered 30-s maximal swimming test (TST). The purpose of this study is to explore gender differences in land and pool-based assessments of anaerobic power (F_peak_) and capacity (F_mean_), as well as the influence of body composition. Thirteen males and fifteen females completed land (Wingate (WAnT)) and pool-based (TST) measures of anaerobic power and capacity previously described in the literature. Additionally, the subjects completed assessments of body composition via air displacement plethysmography. The males produced higher force than the females for F_peak_ (*p* < 0.001) and F_mean_ (*p* = 0.008) during the TST. However, linear regression analysis determined that lean mass significantly predicted F_peak_ (*p* = 0.002) and F_mean_ (*p* < 0.001) during the TST, while gender was no longer significant (*p* = 0.694 and *p* = 0.136, respectively). In conclusion, increases in anaerobic power and capacity (F_peak_ and F_mean_) may be a function of increased lean mass in males and females, warranting future research on the impact of resistance training programs on force production and swimming performance.

## 1. Introduction

Anaerobic power and capacity are important sport-specific fitness components related to overall sports performance and injury prevention [1]. Commonly used anaerobic power tests, such as the Wingate Anaerobic Cycling Test (WAnT), Magaria–Kalamen stair run test, and vertical jump test, have demonstrated consistent differences between males and females, with male subjects producing higher absolute values for peak and mean force [2,3,4,5,6,7,8,9]. However, numerous studies have significantly reduced or eliminated these gender differences in anaerobic performance by expressing power output relative to total body weight and/or fat-free mass [3,4,9,10].

Assessing the anaerobic power and capacity of swimmers presents a unique challenge. With research demonstrating a lack of association between land-based measures of anaerobic power and capacity and swimming performance [11,12], it is well understood that there are a number of factors influencing aquatics-based sports performance (buoyancy, drag, hydrodynamics, etc.). Therefore, recent research has focused on quantifying propulsive forces with direct methods such as the tethered swimming test (TST), which has been shown to be a valid and reliable assessment of anaerobic power and capacity in swimmers [12,13,14,15,16]. Additionally, it has been well established that muscle power production, specifically tethered swimming forces, is associated with and can predict middle-distance and sprint swimming performance, [12,13,17,18,19,20,21,22,23,24,25].

As the TST has grown in popularity over the past decade, so has its application in investigating gender differences in the aquatic environment. While a few studies have consistently reported higher peak and average force production and higher swimming performance (faster swimming speed) in male subjects compared to female [26,27,28,29], the available literature is very limited, as noted in a recent systematic review [16]. Additionally, very few others have sought to examine the influence of body weight and body composition on swimming performance, with no known research investigating the effects of gender, weight, and body composition on force production during a TST. Therefore, the purpose of the current investigation was to examine gender differences in land and pool-based assessments of anaerobic power and capacity. Additionally, this study investigated the influence of body weight and composition on land and pool-based anaerobic power and capacity.

## 2. Materials and Methods

**Figure 1:**
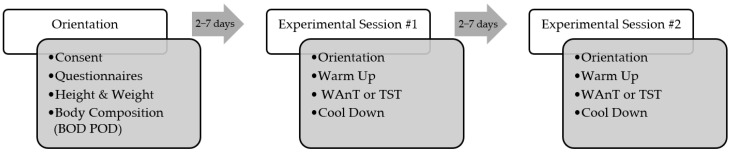
Experimental design scheme.

This study employed a randomized repeated-measures crossover design. Following the appropriate orientations, the subjects completed two experimental sessions: (1) the WAnT and (2) the TST. The order of the experimental sessions was randomized, and they were conducted on separate days with a minimum of two days and a maximum of seven days between sessions (Figure 1). All procedures were approved by the University’s Institutional Review Board.

***Subjects***. Twenty-eight healthy subjects (Male = 13, Female = 15), were included in the study. The subjects were included in the study if they met the following criteria: (1) 18–45 years old, (2) intermediate level swimmer or higher (defined as able to proficiently swim freestyle with rhythmic breathing for 15 min continuously and/or 457.2 m (500 yards)), (3) free of upper or lower extremity musculoskeletal injury or medical condition that would limit their ability to exercise on a cycle ergometer or in shallow water, and (4) comfortable exercising on a cycle ergometer and in shallow water. All subjects completed a medical inventory, the Physical Activity Readiness Questionnaire (PAR-Q) [30], and provided informed consent prior to participation. At baseline, standing height (cm) using a wall-mounted stadiometer (Seca, Hanover, MD, USA), body weight (kg) using an electronic scale (Cosmed, Chicago, IL, USA), and body composition (% body fat (%BF), fat-free mass (FFM), and fat mass (FM)) using the Bod Pod Body Composition System (Cosmed, Chicago, IL, USA) via air displacement plethysmography [31] were assessed. The demographic and anthropometric characteristics of the study participants are presented in Table 1.

***Wingate Anaerobic Test (WAnT).*** Force production on land was evaluated using an electronically braked Velotron cycling ergometer (RacerMate, Inc., Seattle, WA, USA) during the Wingate protocol. This protocol has been well described in the literature and has been shown to be a valid and reliable measure of anaerobic power and capacity [32,33]. After a 5-min warm-up at approximately 125 W, the 30-s Wingate protocol was implemented with a braking force equal to 9.0% body mass for all subjects. The subjects were instructed to pedal as hard and as fast as they could against the applied resistance for the duration of the test, followed by a self-prescribed cool-down.

***Tethered Swimming Test (TST).*** Force production in the water was evaluated using a fully tethered swimming system consisting of a Futek submersible S-Beam load cell, Model LSB210 (Futek, Irvine, CA, USA) firmly attached to a pole at the end of the pool. The load cell was connected to a PC that uses a data acquisition system, SENSIT Test and Measurement (v 2.3.4007.7), with the data sampled at 300 Hz. The load cell was connected to a 2.5 m nonelastic tether and swim belt set at a standardized angle (4°) for each test. The protocol utilized in the current investigation has been previously described in the literature in detail [12,22,34] and has been shown to be a valid and reliable method for measuring anaerobic swimming performance. Following a general warm-up of 500–1000 yards at a self-selected pace, the subjects adopted a horizontal swimming position for 10 s, allowing for adjustment in technique, followed by the 30-s TST, where the subjects were instructed to swim freestyle as hard and as fast as they could while fully tethered for the duration of the test. The TST was performed in the university pool with a consistent water temperature of 26–27 °C. The tethered swimming data were exported to signal processing software (MATLAB R2022a, MathWorks, Natick, MA, USA), and individual force–time curve data were smoothed using a fourth-order Butterworth lowpass digital filter with a cutoff frequency of 4.5 Hz, defined by the residual analysis [35].

***Data Processing.*** The following parameters during the WAnT and the TST were identified for each subject: (a) F_peak_ was defined as the highest force (N) value obtained from the individual force–time curve, and (b) F_mean_ was defined as the average of all force (N) values during the 30-s trial.

***Statistical Analysis.*** Following an assessment of normality (Shapiro–Wilk, independent *t*-tests) were used to determine absolute differences between males and females for F_peak_ and F_mean_ during both the WAnT and TST. Additionally, linear regression analysis with backwards selection was used to determine the influence of gender, body weight, and body composition (%BF, FFM, FM) on F_peak_ and F_mean_ for the WAnT and TST. The level of significance was set a priori at *p* ≤ 0.05.

## 3. Results

The study sample consisted of approximately equal numbers of male and female subjects, and the demographic characteristics are presented in Table 1. While there were no statistical differences in age or body mass index (BMI), the male subjects had higher total body weight and fat-free mass, as well as a lower percentage of body fat than their female counterparts.

Statistically significant differences were observed between the male and female subjects for F_peak_ and F_mean_ in both the land and pool assessments (WAnT and TST), with the male subjects consistently producing higher force than their female counterparts on land and in the water (Table 2).

A stepwise multiple linear regression was calculated to predict F_peak_ and F_mean_ during the WAnT and TST based upon gender, body weight, and body composition. Given that gender was of particular interest, it was retained in all of the models. Preliminary analyses were performed to ensure that there was no violation of the assumption of normality and linearity. Following the removal of all non-significant variables (%BF, FM, total body weight), except for gender, a significant regression equation was found for all dependent variables:

WAnT:

- F_peak_ (N): F(3, 24) = 38.505, *p* < 0.001), with an R^2^ of 0.828;
- F_mean_ (N): F(2, 25) = 58.31, *p* < 0.001), with an R^2^ of 0.823.

TST:

- F_peak_ (N): F(2, 25) = 21.321, *p* < 0.001), with an R^2^ of 0.630;
- F_mean_ (N): F(2, 25) = 14.257, *p* < 0.001), with an R^2^ of 0.533.

**Figure 2:**
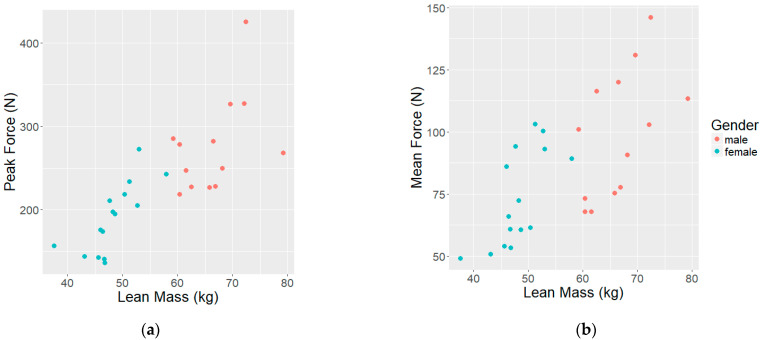
Relationship between lean mass and force production: (**a**) The relationship between peak force production (F_peak_) and lean mass (FFM) during a TST. (**b**) The relationship between average force production (F_mean_) and lean mass (FFM) during a TST.

For all models, it was found that FFM significantly predicted force production (F_peak_ and F_mean_), while gender did not significantly contribute to the prediction model. In addition to FFM, %BF significantly contributed to F_peak_ during the WAnT. In summary, it was found that FFM was the best predictor of force production during the TST, which accounted for 63% and 53% of the variance in F_peak_ and F_mean_, respectively. Additionally, for every 1 kg of lean mass, a subject produced 5.39 N higher F_peak_ and 2.596 N higher F_mean_ during the TST. The results of the analysis can be found in Table 3, and the relationship between force production and lean mass is shown in Figure 2.

## 4. Discussion

The aim of this study was to examine gender differences and the influence of body weight and composition on peak and average force production during land and pool-based assessments of anaerobic power and capacity. It has been well established that males have higher absolute power output than females in land-based assessments of anaerobic power and capacity [2,3,4,5,6,7,8,9]. The present investigation adds further evidence to this claim, showing that males produced higher peak and average force during a WAnT. The results of this study also suggest that gender is not a significant predictor of force production during the WAnT. Additionally, the findings indicate a linear association between lean mass and peak and average power output, which is consistent with other research [3,4,7,9,10].

However, gender differences in force production in the pool have received limited attention in the literature. In a recent systematic review, Santos and colleagues referenced only four studies analyzing propulsive forces between genders [16]. Building on and concurring with the findings of these previously published studies [26,27,28,29], the current investigation showed differences between males and females for absolute peak and mean force production during a pool-based anaerobic assessment (TST), with males producing higher forces than females. As previously stated, force production in the water is associated with swimming performance [12,13,17,18,19,20,21,22,23,24,25]. While previous publications have explained the gender-based performance gap by the interactions between motor control and anthropometric and kinematic features [36], the literature appears to suggest that male swimmers are faster mostly due to their ability to generate higher propulsive forces [16,27].

Of particular interest, the current investigation sought to determine the influence of gender, body weight, and body composition on force production in the water. Similar to land-based findings, the results of this study also demonstrate that gender does not significantly contribute to predicting force production in the water during the TST. Alternatively, the data show that force production in the water was significantly influenced by the subject’s lean mass. As such, the results of the regression analysis indicate that the peak and mean power output increases linearly with the amount of lean mass. Given that the female subjects had significantly lower lean mass than their male counterparts, it can be strongly suggested that the main reason for the gender difference in absolute peak and mean force production during the TST is simply that the female subjects had less muscle mass. While this has previously not been directly studied in swimming, the results of this analysis are consistent with the findings of land-based studies [3,4,7,9,10]. Additionally, some have concluded that swimming performance, which is highly correlated to force production [12], is influenced by body size. Thus, it has been suggested that if a larger body size results in better performance, then females’ slower swimming times are due to their smaller size compared to males [37,38]. The results of the current investigation build on this conclusion, by specifically identifying that increased lean mass, rather than just overall body size, may influence performance via increased force production. However, more research should be done to determine the concurrent influence of body size and composition with the inclusion of additional anthropometric measurements, such as height, segment (arm and leg) length, and hand size.

Given this relationship between lean mass, force production, and swimming performance, it may be inferred that increasing lean mass with a well-designed resistance training program would result in improved sprint swimming performance. The TST is a stationary swim test with no forward motion, and therefore, it may change swimming kinematics. It also does not account for the counteracting effects of increased drag forces and/or decreased buoyancy associated with changes in muscle mass and body composition during free swimming, as has been well described in the literature [16,36,37]. As such, the benefits of a well-designed resistance training program for swimmers have been previously reviewed [39,40], although it has been suggested that coaches have concerns with certain styles of resistance training programs due to the aforementioned potential to increase muscle mass (hypertrophy) or decrease flexibility, which would increase drag forces and negatively influence swimming performance [41,42]. Even so, a recent systematic review by Crowley et al. summarized the available literature, suggesting that specific low-volume, high-velocity training programs are optimal for improving swimming performance [41]. However, there is a lack of consistency in program design and limited longitudinal research on the effects of a resistance training program on increased muscle mass, force production, and ultimately, swimming performance.

While the aim of the current investigation was to examine the influence of gender and body composition on force production, several limitations should be noted. First, the current investigation included a relatively small sample size with a broad spectrum of inclusion criteria. While this heterogeneous sample may lend to the generalizability of the results, the influence of age, training status, and level of swimming expertise on kinematics, force production, and swimming performance has been well established [16,36]. Therefore, future studies should seek to examine the influence of gender and body composition in a larger sample while investigating and controlling for the aforementioned characteristics, specifically, training level and swimming kinematics. Additionally, future research should seek to address the limitations of this and previous studies by comprehensively investigating the relationship between force production, swimming speed, and performance and anthropometric measures, including body weight, size, body composition, and lean mass. Additionally, future research should aim to develop high-quality methodological studies using elite swimmers to investigate the effects of a well-designed hypertrophic resistance training program of adequate duration (12 weeks or more) on body weight, body composition, lean muscle mass, muscle cross-sectional area, tethered swimming force production, and the transference to swimming performance.

## 5. Conclusions

In summary, the results of this study indicate that while gender differences exist for absolute force production during land and water-based assessment, the main factor accounting for gender differences in peak and mean force production during a TST is the amount of lean mass. From this, it can be concluded that resistance training programs designed to increase lean muscle mass may potentially benefit sprint swimming performance, although more research is needed to better understand the optimal body weight or lean mass to power ratio in swimming.

## Figures and Tables

**Table 1:** Demographic characteristics.

Variables	Males ^1^	Females ^1^	Total ^1^	*p*-Value
Age (years)	22.54 ± 3.07	20.67 ± 4.46	21.54 ± 3.93	0.215
Weight (kg)	77.53 ± 7.62	65.36 ± 8.11	71.01 ± 9.91	<0.001 **
Body Mass Index (kg/m^2^)	24.63 ± 2.45	23.36 ± 2.93	23.95 ± 2.75	0.229
Percent Body Fat (%BF)	13.92 ± 5.13	25.80 ± 7.94	20.28 ± 8.99	<0.001 **
Fat-Free Mass (FFM) (kg)	66.49 ± 5.83	48.10 ± 4.71	56.64 ± 10.67	<0.001 **
Fat Mass (FM) (kg)	10.97 ± 4.38	17.25 ± 6.8	14.33 ± 6.54	0.008 *

^1^ Data presented as mean ± SD; * *p* < 0.05; ** *p* ≤ 0.001.

**Table 2:** Land and pool-based force production gender differences.

	Males ^1^	Females ^1^	Total ^1^	*p*-Value
WAnT				
F_peak_ (N)	1000.69 ± 165.88	724.87 ± 104.12	852.93 ± 193.58	<0.001 **
F_mean_ (N)	707.61 ± 96.54	443.27 ± 96.41	566.00 ± 164.28	<0.001 **
TST				
F_peak_ (N)	277.63 ± 56.72	193.79 ± 37.20	230.06 ± 65.41	<0.001 **
F_mean_ (N)	99.30 ± 25.59	73.45 ± 19.23	84.92 ± 25.65	0.005 *

^1^ Data presented as mean ± SD; * p < 0.05; ** p ≤ 0.001.

**Table 3:** The influence of gender and body composition on land and pool-based force production.

	β	β_std_	*p*-Value
WAnT F_peak_			
Gender	−48.728	−0.128	0.488
FFM	18.828	0.466	<0.001 **
%BF	10.039	5.815	0.001 **
WAnT F_mean_			
Gender	−33.299	−0.103	0.559
FFM	12.561	0.816	<0.001 **
TST F_peak_			
Gender	12.913	0.100	0.694
FFM	5.396	0.880	0.002 *
TST F_mean_			
Gender	21.996	0.435	0.136
FFM	2.596	1.080	<0.001 **

* p < 0.05; ** *p* ≤ 0.001.

## Data Availability

The data presented in this study are available on request from the corresponding author. The data are not publicly available due to privacy.
